# Generation of an algorithm based on minimal gene sets to clinically subtype triple negative breast cancer patients

**DOI:** 10.1186/s12885-016-2198-0

**Published:** 2016-02-23

**Authors:** Brian Z. Ring, David R. Hout, Stephan W. Morris, Kasey Lawrence, Brock L. Schweitzer, Daniel B. Bailey, Brian D. Lehmann, Jennifer A. Pietenpol, Robert S. Seitz

**Affiliations:** Institute of Personalized and Genomic Medicine, College of Life Science, Huazhong University of Science and Technology, Wuhan, China; Insight Genetics Incorporated, Nashville, Tennessee USA; Department of Biochemistry, Vanderbilt-Ingram Cancer Center, Vanderbilt University School of Medicine, Nashville, Tennessee USA

**Keywords:** Translational oncology, Breast cancer, Biomarkers, Statistical analysis

## Abstract

**Background:**

Recently, a gene expression algorithm, TNBCtype, was developed that can divide triple-negative breast cancer (TNBC) into molecularly-defined subtypes. The algorithm has potential to provide predictive value for TNBC subtype-specific response to various treatments. TNBCtype used in a retrospective analysis of neoadjuvant clinical trial data of TNBC patients demonstrated that TNBC subtype and pathological complete response to neoadjuvant chemotherapy were significantly associated. Herein we describe an expression algorithm reduced to 101 genes with the power to subtype TNBC tumors similar to the original 2188-gene expression algorithm and predict patient outcomes.

**Methods:**

The new classification model was built using the same expression data sets used for the original TNBCtype algorithm. Gene set enrichment followed by shrunken centroid analysis were used for feature reduction, then elastic-net regularized linear modeling was used to identify genes for a centroid model classifying all subtypes, comprised of 101 genes. The predictive capability of both this new “lean” algorithm and the original 2188-gene model were applied to an independent clinical trial cohort of 139 TNBC patients treated initially with neoadjuvant doxorubicin/cyclophosphamide and then randomized to receive either paclitaxel or ixabepilone to determine association of pathologic complete response within the subtypes.

**Results:**

The new 101-gene expression model reproduced the classification provided by the 2188-gene algorithm and was highly concordant in the same set of seven TNBC cohorts used to generate the TNBCtype algorithm (87 %), as well as in the independent clinical trial cohort (88 %), when cases with significant correlations to multiple subtypes were excluded.

Clinical responses to both neoadjuvant treatment arms, found BL2 to be significantly associated with poor response (Odds Ratio (OR) =0.12, *p* =0.03 for the 2188-gene model; OR = 0.23, *p* < 0.03 for the 101-gene model). Additionally, while the BL1 subtype trended towards significance in the 2188-gene model (OR = 1.91, *p* = 0.14), the 101-gene model demonstrated significant association with improved response in patients with the BL1 subtype (OR = 3.59, *p* = 0.02).

**Conclusions:**

These results demonstrate that a model using small gene sets can recapitulate the TNBC subtypes identified by the original 2188-gene model and in the case of standard chemotherapy, the ability to predict therapeutic response.

## Introduction

TNBC has a higher rate of 5-year distant recurrence than other breast cancers, and despite adjuvant chemotherapy as standard of care for this cancer, 5-year recurrence rates are around 30 % [1]. Those patients that achieve a pathological complete response (pCR) to neoadjuvant chemotherapy have significantly better overall survival [1, 2]. Furthermore, the correlation between pCR and distant recurrence is considerably stronger within TNBC patients compared to ER+ patients [3] leading the Food and Drug Administration to allow pCR as a clinical endpoint for TNBC while strongly recommending against it in ER+ patients [4]. Many studies have established that breast tumors are heterogeneous, both in histology and clinical outcome, and these differences can serve as the basis for clinical classification and prognostication [5]. Additionally, molecular classification of cancer subtypes is becoming an increasingly important tool in devising treatment plans. For example, mutation analysis of KRAS in colorectal cancer [6], and EGFR mutation and ALK rearrangement detection in non-small cell lung cancer [7, 8] are now standard of care.

There currently exist no clinically applied molecular subclassification tools for TNBC. The intrinsic breast cancer classification system [9], which has proven useful in assigning biological information to breast cancer subclasses, categorizes the majority of TNBC cases within the basal subclass [10]. However, significant heterogeneity – both clinically and pathologically – exists in TNBC, and better subclassification tools are needed for clinical decision-making. To this end, Lehmann et al. used 21 breast cancer data sets containing 587 TNBC cases and employed cluster and gene expression analysis to identify a set of 2188 genes for the classification of TNBC into six subtypes (two basal-like (BL1 and BL2), immunomodulatory (IM), mesenchymal (M), mesenchymal stem–like (MSL), and luminal androgen receptor (LAR) subtypes) [11]. The goal is of this study to translate the knowledge of biologically distinct subtypes into rational design of pre-clinical studies for TNBC clinical trials and to facilitate the identification of novel predictive markers. A previous study has shown the promise of clinical utility by retrospectively subtyping 130 TNBC patients who had received standard neoadjuvant chemotherapy comprised of anthracycline, cyclophosphamide and a taxane. This study showed that patients with basal-like BL1 tumor subtypes had an improved response (52 % exhibiting pCR) whereas basal-like BL2 tumor subtypes showed a worse response to standard chemotherapy (0 % pCR) [12].

In the derivation of the TNBCtype subclassification tool, the final group of 2188 classifying genes was identified from an initial set of approximately 13,000 genes by selection of those genes with expression significantly distinct from the median gene expression among all the cluster-defined subclasses. Although a seminal advance for the TNBC field, this large classification panel could best be applied for clinical use only after further refinement. Such optimization would serve three purposes. First, a more limited set of genes would speed translation of the classifier into a cost-effective clinical tool. Second, although not necessarily the case [13], the genes most predictive of a subtype may include those most relevant to the regulation and function of that subtype; therefore, a smaller set of genes may increase the likelihood of correlating biological meaning to the panel members and allowing easier comparison of TNBC subtype molecular profiles to other similarly well-defined molecular prognostic and predictive tools. Third, and most importantly, a small set of classifying genes could improve the reproducibility of the TNBC subtyping panel.

Initial gene expression analysis often has the problem of inclusion of genes with little signal contribution. This problem arises from having tens of thousands of genes produced in a typical assay but a considerably smaller number of measured samples within which to assess these potential classifiers. This statistical problem, coupled with the inherent noise of microarray platforms, creates a challenge to the derivation of reproducible classification panels. One study estimated that to achieve similar (i.e., overlapping) gene panels in multiple cohorts, the number of analyzed tumor samples would need to be at least several thousand [14]. It is well established that overfitting occurs with large-scale gene expression data when poor or no feature or dimensional reduction is attempted [15, 16]. Careful reduction of the genes included in a TNBC classifier would potentially lead to a robust clinically applicable tool for subtype identification.

Here we describe the development and validation of a new TNBC classification tool using only 101 genes, less than 5 % of the size of the original 2188 gene model of TNBCtype and yet able to reproduce its classification. The association of the BL1 and BL2 subtypes with pathologic response was also reproduced in an independent patient cohort using this new model.

## Methods

### Gene expression data set processing

Twenty-one expression data sets representing 13,060 unique genes that were previously used to develop (14 data sets; *N* = 386 patients) and validate (7 data sets; *N* = 201 patients) the 2188-gene TNBC classification model were used as prepared for the published analysis (Robust Multi-array Average (RMA) normalized, log transformed) [11]. Expression data of an additional breast cancer data set (TNBC: GSE41998) was downloaded along with clinical metrics including age, menopausal status, and outcome as measured by pCR. Patient datasets were previously made publicly available under the ethical policies of the National Institutes of Health’s GEO database (http://www.ncbi.nlm.nih.gov/geo/info/submission.html). No additional ethics review was required for the *in silico* analysis of these data sets.

As with the Lehmann et al*.* analysis, when multiple probes for a gene were present, the probe with the highest inter-quartile range was selected. Triple-negative status in the GSE41998 breast cancer samples was determined by the given pathological diagnosis (*N* = 139 patients), with an additional seven cases being excluded because bimodal modeling of ER, PR, and ERBB2 expression gave posterior probabilities greater than 0.5 that these genes were expressed. The original 2188-gene centroid classifier, five individual subtype classification models, and a 101-gene centroid classifier were applied to this patient set.

### Statistical analysis and model building

The TNBC subtype signatures from the original 2188-gene model were used to identify gene sets that distinguished the classes via gene set enrichment analysis (GSEA) using the C2 curated gene sets of canonical pathways [17]. Linear regression, targeted maximum likelihood estimation [18], random forest [19], and elastic-net regularized linear models [20] were employed to create subclassifying models, with each subclass (i.e., BL1, BL2, LAR, M, MSL, and IM) being defined by an individual model. Strength of association with outcome variables was assessed using logistic regression and the Fisher exact test. Classification error was estimated using a bootstrap analysis, and the elastic net models showed the least error (average disagreement of 6 % for all five models). The genes that contribute to the five individual subtype models were combined to create a 101-gene centroid model. All model coefficients and cutoffs were determined using the 14 discovery data sets, as in the original Lehmann et al*.* analysis, and were not altered afterwards. Pathway analysis of the 258 shrunken centroid defined genes was performed with Cytoscape using the ClueGO tools [21, 22]. All *p*-values are two-sided.

## Results

### Building limited gene models of TNBC subtypes

The expression data sets used to generate the original 2188-gene model were assigned TNBC subclass identities based on the Lehmann et al*.* results. Gene set enrichment analysis [17] was performed on the 14 training gene sets and 5639 genes were identified as belonging to pre-defined gene sets that associate with the TNBC subclasses. Given previous observations that tumor infiltrating lymphocytes (TILs) correlate with increased expression of genes involved in immune response [23], the ‘Immunomodulatory’ (IM) subtype likely reflects the presence of gene expression contributed by immune infiltrates with the tumor cells having the signature of a different subtype. Therefore we performed principal component analysis (PCA) to identify and remove the IM component. The presence of an IM component almost completely defined the IM class (data not shown), and its significant association with other classes caused a significant loss of information. Therefore, cases assigned an IM identity were excluded and analyzed separately.

**Fig. 1:**
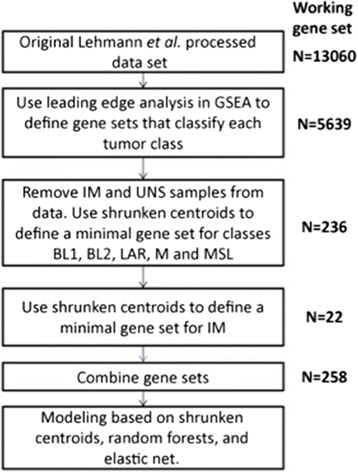
Gene selection process for model building. Creation of a minimal gene set employed gene set enrichment, shrunken centroid analysis, and modeling using shrunken centroids, random forests, and elastic nets

Additionally, cases not classified by the original TNBCtype were also excluded, as well as cases that a Z-test showed to have non-significant differences between the most highly correlated centroids. Shrunken centroid analysis [24] was used for further feature reduction. Using all non-IM cases, 236 genes were identified as likely classifiers. Analyzing the IM cases compared to all other combined cases identified a further 22 gene classifiers, resulting in 258 genes in total used for subsequent model building (Fig. 1).

**Fig. 2:**
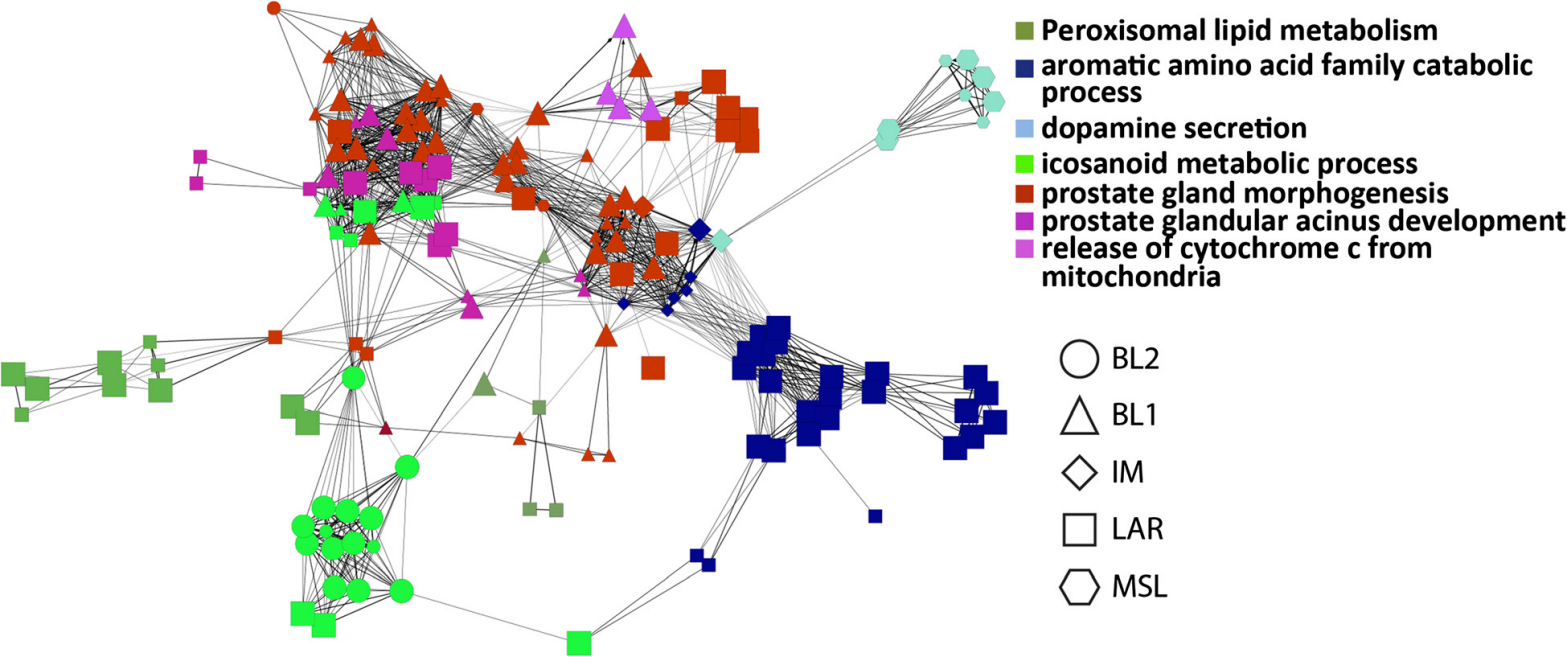
Pathway analysis of GSEA-defined classifying genes. The 258 genes used for model building were mapped to KEGG pathways and GO biological processes, and the network created from these functional groups was then viewed. The network is color coded by the KEGG and GO terms and the TNBC subtype associated with the genes are designated by the shape of the network nodes

Pathway analysis of the shrunken centroid-defined list of 258 genes used for model building and their associated GO and KEGG terms showed biological processes consistent with their putative classification role, which lent confidence to this limited gene list (Fig. 2). Different gene sets and algorithms were used for the initial gene set enrichment and this pathway analysis, and no supervision was employed over pathways used to define subtypes. As an example, most of the genes associated with the BL1 subclass correlated with the expression of genes previously observed in basal cells [25]. Additionally, genes associated with the LAR subclass mapped to clusters of peroxisomal lipid metabolism and aromatic acid metabolism and catabolism, which matches the functions previously mapped to this subtype [10].

Linear regression, targeted maximum likelihood estimation [18], random forest [19], and elastic-net regularized linear models [20] were employed to create subclassification models, with the latter approach giving the best fit to the TNBCtype-designated subclasses with the least number of required genes. Six elastic net models were created to identify each subtype individually, or an expression-based centroid defined by the genes used in all the elastic net models—101 genes in total. While the 101 genes were selected independently of the original 2188 genes, 96 genes were in common between the two models*.* These models were then applied to the set of seven, validation cohorts employed in the TNBCtype analysis. The elastic net-defined models showed a predicted misclassification rate of 2–9 % in the discovery set of cohorts in a bootstrap analysis, and 6–17 % in the validation cohorts (Table 1). The 101-gene centroid had a 7 % discordance in the discovery cohort, while in the validation cohort the discordance was 25 % (Table 2A). The centroid model allows a tumor to be assigned to only one subclass, in contrast to the individual models, though some cases show characteristics of multiple subtypes. When cases were excluded that showed a significant correlation to multiple subtypes and an insignificant (via a Z-test) difference between correlation to these subclasses, the discordance in the validation cohort decreased to 13 % (Table 2B). As the 101 gene centroid model did not require the use of fixed cutoff to classify samples and was thus platform independent, this model was used for the subsequent analyses of this investigation.

**Table 1:** Misclassification rate

	Lehmann et al*.* discovery set (*N* = 386)	Lehmann et al*.* validation set (*N* = 201)
BL1	0.07	0.14
BL2	0.06	0.06
LAR	0.02	0.12
M	0.09	0.17
MSL	0.04	0.10

Misclassification rate as estimated by bootstrap analysis of elastic net models in the Lehmann et al*.* [11] discovery and validation cohorts

**Table 2:** Comparison of 2188- and 101-gene centroid classifiers in the Lehmann et al*.* [11] validation cohorts

			TNBCtype (2188 gene centroid model)						
			BL1	BL2	LAR	M	MSL	IM	Unclassified
101 gene centroid model	(A) All significantly classed cases	BL1	25			1	3	16	4
		BL2	1	15	1	2	1	7	2
		LAR		1	14	3	1		
		M	6	1	1	26	2		2
		MSL	2	1	2	4	17	5	2
		Unclassified	2	5		6	1	16	3
	(B) Ambiguous cases unclassified	BL1	19				2	14	3
		BL2	1	12				7	2
		LAR			13	1	1		
		M	2		1	21	1		1
		MSL	1			2	14	4	2
		Unclassified	13	11	4	18	7	19	5

In panel A, all cases are used. In Panel B, ambiguous cases—cases that showed an insignificant difference (via a Z-test) between subclasses—are placed in the unclassified group

### Comparison of the original and newly developed subtyping models in an independent cohort of patients treated with doxorubicin/cyclophosphamide followed mitotic inhibitors

A previous study had demonstrated that TNBC molecular subtypes differed in response to neoadjuvant chemotherapy [12]. To determine if subtype classifications provided by TNBCtype and the newly developed limited gene models were concordant, we evaluated an independent cohort of 278 early-stage breast cancer patients, of which 139 were TNBC patients, treated neoadjuvantly with doxorubicin/cyclophosphamide (AC) followed by ixabepilone or paclitaxel [26]. Agreement between the 2188-gene and the 101-gene centroid classifiers was 83 % among cases that had a significant correlation with at least one subtype. When the comparison was limited to those samples that had a significant difference in the correlations between the highest and second highest subtype, the agreement increased to 86 % (Table 3).

**Table 3:** Comparison of 2188- and 101-gene centroid classifiers in the GSE41998 TNBC cohort [26]

			TNBCtype (2188 gene centroid model)					
			BL1	BL2	LAR	M	MSL	Unclassified
101-gene centroid model	(A) All significantly classed cases	BL1	36			1	1	
		BL2	5	12		2	2	
		LAR		1	10		1	1
		M	3	1		25	2	
		MSL				3	21	
		Unclassified	1		1	1	1	1
	(B) Ambiguous cases unclassified	BL1	26					
		BL2	5	11		1	2	
		LAR		1	9			1
		M		1		15	1	
		MSL				1	11	
		Unclassified	14	1	2	15	14	1

In panel A, all cases are used. In Panel B, ambiguous cases—cases that showed an insignificant difference (via a Z-test) between subclasses—are placed in the unclassified group

### BL1 and BL2 TNBC subtypes differ in pathologic response to mitotic inhibitors

The clinical response of TNBC patients in the GSE41998 cohort to neoadjuvant AC was strongly predictive of pCR after subsequent additional treatment with ixabepilone or paclitaxel (*p* < 0.0001, Table 4). Age was also a very strong predictor of outcome, as was menopausal status (Table 5). Stage was not included in the provided data set and could therefore not be assessed as a factor. *Also not provided was BRCA1/2 status, a factor previously shown to be predictive of response to certain chemotherapeutic agents* [27]. In previous cohorts, patients with BL1 subtype tumors had better response to chemotherapy and those with BL2 had lower rates of response [12], and similar results were found in this cohort. Patients with BL2 subclass tumors, as defined by either the 2188- or 101-gene models (with confounding calls removed for both cases as described above), had the least response and higher incidence of more than minimal residual disease after therapy (OR = 0.12, *p* = 0.03; OR = 0.23, *p* = 0.02 for the 2188- and 101-gene models, respectively, via a Fisher exact test) (Table 6). The BL1 subclass as defined by the 101-gene model exhibited the best response (OR = 3.59, *p* = 0.02). The direction of association of BL1 with pCR or minimal residual cancer burden (RCB) in the 2188-gene model was similar (OR = 1.91) but did not reach significance (OR = 0.14). When all cases were examined with the 101-gene model (i.e., including cases that mapped to multiple subtype but were assigned to the subtype with the highest correlation), the BL2 subtype retained a significant association with poor response to therapy while BL1 cases lost significance. When the 2188-gene model was analyzed in this manner, neither BL1 nor BL2 had a significant association with response to therapy, though the effect sizes were similar (data not shown *Whether this is due to superior performance by the 101-gene algorithm or a due to the small size of the cohort cannot be determined without further study.*

**Table 4:** Comparison of clinical response

		pCR/mRCB		pCR	
		No	Yes	No	Yes
Clinical Response to AC	complete response	5	18	8	15
	partial response	36	31	39	28
	stable disease	20	2	20	2
	progressive disease	2	0	2	0

Clinical response to neoadjuvant AC (doxorubicin/cyclophosphamide) and pCR)/minimal RCB after subsequent neoadjuvant ixabepilone or paclitaxel in the GSE41998 TNBC cohort [26]

**Table 5:** Clinical variables with association to outcome

		AC response		pCR/RCB	
		T score	*P* value	score	*P* value
Univariate analysis	age	-2.71	0.007	-2.1	0.036
	tumor size	-0.29	0.768	-1.08	0.28
	menopausal status	-3.41	0.001	-1.52	0.127
Multivariate analysis	age	-0.32	0.749	-2.29	0.022
	tumor size	-0.97	0.331	-2.15	0.032
	menopausal status	-2.29	0.022	0.7	0.485

Association of clinical variables with outcome as measured by logistic regression in the GSE41998 TNBC cohort

**Table 6:** Association of centroid model-determined subtype and pCR

		pCR/mRCB				
		Yes	No	Percentage	Odds Ratio	*p* value
2188 gene centroid	BL1	20	14	59 %	1.91	0.14
	BL2	1	8	11 %	*0.12*	*0.03*
	LAR	3	6	33 %	0.5	0.49
	M	7	12	37 %	0.56	0.31
	MSL	15	9	63 %	2.13	0.16
	Unc	7	15	32 %		
101 gene centroid	BL1	16	7	70 %	*3.59*	*0.02*
	BL2	4	14	22 %	*0.23*	*0.02*
	LAR	3	6	33 %	0.5	0.48
	M	7	7	50 %	1.1	0.99
	MSL	6	5	55 %	1.35	0.75
	Unc	17	25	40 %	NA	NA

Cases with significant association with more than one subclass were excluded. *P* values determined by Fisher Exact test

## Discussion

TNBC comprises up to 20 % of all breast cancers (as many as 40,000 women newly diagnosed in the US each year), and occurs more frequently in young and African-American women [1]. TNBC has higher rates of metastatic recurrence and poorer prognosis than other breast cancers, with a 5-year survival of only ~70 % after treatment with the most aggressive conventional cytotoxic chemotherapies. This current state is due in large part to the heterogeneity of TNBC and the still limited knowledge regarding therapeutic targets and biomarkers that can predict the responsiveness of these cancers to either standard-of-care or investigational therapies. Despite overall poor outcomes, approximately 30 % of TNBC patients respond to standard chemotherapy [1]. Thus, there is a critical unmet need to develop focused diagnostics to identify patients that would benefit from standard chemotherapy and better align new therapeutic regimens with actionable targets expressed in TNBC patients. The TNBC subtype algorithm represents a major advance toward addressing the heterogeneity and therapeutic sensitivities of TNBC [11]. However, certain features of this original algorithm, such as the large number of genes that comprise it (2188 in total), are not optimal for its routine clinical application. The refinements described herein represent a portion of the optimization steps being performed to ultimately offer TNBC subtyping as a test with clinical utility.

Bioinformatics refinement of the original, academic research-based TNBCtype algorithm allowed minimization of the expression signature representative of all of the TNBC subtypes from 2188 to only 101 genes. Importantly, there was excellent agreement between the originally proposed 2188-gene subclassification model and the new “lean” 101-gene classifier in both a set of discovery and validation TNBC cohorts as well as in an independent TNBC clinical trial cohort treated neoadjuvantly with AC followed by the mitotic inhibitors [26]. The gene set enrichment analysis that allowed the pruning of the original model of 2188 genes into only 101 genes showed comparable classification and predictive utility. The data suggest that in the 101-gene model, the genes that define each subclass have similar biological function (Fig. 2). Further, from a practical standpoint, the reduction of the classifier to 101 genes with definition of the individual TNBC subtypes by only 8 to 15 genes will allow placement on assay platforms that would be technically challenging or impossible for the 2188-gene signature.

Preliminary evidence suggestive of the clinical utility of TNBC subtyping has already been demonstrated for both the original 2188-gene and the optimized 101-gene models. In the clinical trial cohort [26] analyzed herein using both models, the BL2 subtype was demonstrated to significantly associate with lack of tumor response to standard chemotherapy, whereas the BL1 subtype significantly associated pCR. Age was a significant predictor of pathological responses in this cohort, but the BL1 and BL2 subtypes (as defined by the 101-gene model) were independent of this factor. To put these findings in context and emphasize their potential relevance to clinical management, it is important to note that historical data show only approximately 25 % of TNBC patients will respond with pCRs to the conventional anthracycline/cyclophosphamide/mitotic inhibitor combination chemotherapy used as neoadjuvant treatment in the test cohort [28, 29]. By subclassifying a TNBC population with the 101-gene model, we found that 70 % of patients with tumors classified as BL1 experienced pCR, in contrast to only 22 % of those with BL2 tumors. Our findings corroborate the independent study published by Masuda et al., who employed the 2188-gene model on a cohort of patients from the MD Anderson Cancer Center treated with neoadjuvant chemotherapy containing sequential taxane and anthracycline-based regimens and likewise found BL1 TNBC patients to have a high rate of pCR (52 %) and BL2 patients to have the lowest (0 %) pCR rate of all subtypes [12]. Collectively, these data are supportive not only of the ability of the gene expression models to classify TNBC into stable homogenous subtypes, but also of the likely predictive utility of these subtypes to assess therapeutic sensitivities.

In the original identification of the TNBC subtypes by Lehmann and co-workers, it was noted that the BL1 subtype was typified by high expression of cell cycle and DNA damage response genes [11]. Additionally, TNBC cell lines that shared expression patterns with this subtype preferentially responded to cisplatin and it was hypothesized that patients with BL1 would have higher response rates to platinum compounds and PARP inhibitors [27] compared to the other subtypes [11]. The 101-gene model is being further refined to an even more limited gene sets to individually classify each subtype. Thereafter, clinical utility studies will follow to assess the ability of subtyping to guide therapeutic decisions regarding the use of platinum agents, PARP inhibitors, as well as other agents believed to have efficacy in subsets of TNBC patients (e.g., checkpoint blockade inhibitors, androgen receptor antagonists and anti-angiogenics such as bevacizumab, etc.). Previous attempts with targeted therapies in unselected TNBC have largely been unsuccessful as has been the case with VEGFR and EGFR inhibitors [30, 31]. However, alignment of targeted therapies with select subsets of TNBC that display biologies dependent on a given target may accelerate development of new therapeutics that are more efficacious for patients with TNBC.

## Conclusion

Our results demonstrate that a model using small gene sets can recapitulate the TNBC subtypes identified by the original 2188-gene model and in the case of standard chemotherapy, the ability to predict therapeutic response. *Additional studies are planned comparing both models on randomized clinical trial samples to fully explore the utility of models to identify responsive patient populations.*

## Abbreviations

TNBC: Triple-negative breast cancer
OR: Odds ratio
pCR: Pathologic complete response
ER: Estrogen receptor
KRAS: Kirsten rat sarcoma viral oncogene homolog
EGFR: Epidermal growth factor receptor
ALK: Anaplastic lymphoma tyrosine kinase receptor
BL1: Basal like 1
BL2: Basal like 2
IM: Immunomodulatory
M: Mesenchymal
MSL: Mesenchymal stem like
LAR: Luminal androgen receptor
RMA: Robust multi-array average
PR: Progesterone receptor
ERBB2: erb-b2 receptor tyrosine kinase
GSEA: Gene set enrichment analysis
TILs: Tumor-infiltrating lymphocytes
PCA: Principal component analysis
AC: Anthracycline/cyclophosphamide
RCB: Residual cancer burden
