# Erratum to: Generation of an algorithm based on minimal gene sets to clinically subtype triple negative breast cancer patients

**DOI:** 10.1186/s12885-016-2307-0

**Published:** 2016-04-18

**Authors:** Brian Z. Ring, David R. Hout, Stephan W. Morris, Kasey Lawrence, Brock L. Schweitzer, Daniel B. Bailey, Brian D. Lehmann, Jennifer A. Pietenpol, Robert S. Seitz

**Affiliations:** Institute of Personalized and Genomic Medicine, College of Life Science, Huazhong University of Science and Technology, Wuhan, China; Insight Genetics Incorporated, Nashville, Tennessee USA; Department of Biochemistry, Vanderbilt-Ingram Cancer Center, Vanderbilt University School of Medicine, Nashville, Tennessee USA

## Erratum

Since publication of our article [1], we have noticed an error in labelling the p-value of one of the results. The original document read:

“The direction of association of BL1 with pCR or minimal residual cancer burden (RCB) in the 2188-gene model was similar (OR = 1.91) but did not reach significance (OR = 0.14).”

This has been corrected to:

“The direction of association of BL1 with pCR or minimal residual cancer burden (RCB) in the 2188-gene model was similar (OR = 1.91) but did not reach significance (p = 0.14).”

We regret the error.
